# Overwintering Distribution of Fall Armyworm (*Spodoptera frugiperda*) in Yunnan, China, and Influencing Environmental Factors

**DOI:** 10.3390/insects11110805

**Published:** 2020-11-15

**Authors:** Yanru Huang, Yingying Dong, Wenjiang Huang, Binyuan Ren, Qiaoyu Deng, Yue Shi, Jie Bai, Yu Ren, Yun Geng, Huiqin Ma

**Affiliations:** 1 Key Laboratory of Digital Earth Science, Aerospace Information Research Institute, Chinese Academy of Sciences, Beijing 100094, China; huangyanru19@mails.ucas.ac.cn (Y.H.); renyu@aircas.ac.cn (Y.R.); gengyun17@mails.ucas.ac.cn (Y.G.); mahq@aircas.ac.cn (H.M.); 2 University of Chinese Academy of Sciences, Beijing 100049, China; baijie19@mails.ucas.ac.cn; 3 National Agricultural Technology Extension and Service Center, Beijing 100125, China; renbinyuan@agri.gov.cn; 4 School of Geographic Sciences, East China Normal University, Shanghai 200241, China; 51193901021@stu.ecnu.edu.cn; 5 Key Lab. of Geographic Information Science (Ministry of Education), School of Geographic Sciences, East China Normal University, Shanghai 200241, China; 6 Department of Computing and Mathematics, Manchester Metropolitan University, Manchester M1 5GD, UK; Y.Shi@mmu.ac.uk; 7 State Key Laboratory of Remote Sensing Science, Aerospace Information Research Institute, Chinese Academy of Sciences, Beijing 100101, China

**Keywords:** fall armyworm, overwintering, pests, potential distribution, suitability

## Abstract

**Simple Summary:**

The fall armyworm (*Spodoptera frugiperda*) is a nondiapausing insect pest capable of causing large reductions in the yield of crops, especially maize. Every year, the new generation of fall armyworms from Southeast Asia flies to East Asia via Yunnan, and some of them will grow, develop and reproduce in Yunnan since the geographical location and environmental conditions of Yunnan are very beneficial for the colonization of fall armyworms. This study explored the potential overwintering distribution of fall armyworms in Yunnan and the influence of environmental factors on its distribution. These results provide a basis for the precise prevention and control of fall armyworms by guiding management and decision-making and may facilitate meaningful reductions in pesticide application.

**Abstract:**

The first fall armyworm (FAW; *Spodoptera frugiperda*) attack in Yunnan, China, occurred in January 2019. Because FAW lacks diapause ability, its population outbreaks largely depend on environmental conditions experienced during the overwinter months. Thus, there is an urgent need to make short-term predictions regarding the potential overwintering distribution of FAW to prevent outbreaks. In this study, we selected the MaxEnt model with the optimal parameter combination to predict the potential overwintering distribution of FAW in Yunnan. Remote sensing data were used in the prediction to provide real-time surface conditions. The results predict variation in the severity and geographic distribution of suitability. The high potential distribution shows a concentration in southwestern Yunnan that suitability continues to increase from January to March, gradually extending to eastern Yunnan and a small part of the northern areas. The monthly independent contributions of meteorological, vegetation, and soil factors were 30.6%, 16.5%, and 3.4%, respectively, indicating that the suitability of conditions for FAW was not solely dominated by the weather and that ground surface conditions also played a decisive role. These results provide a basis for the precise prevention and control of fall armyworms by guiding management and decision-making and may facilitate meaningful reductions in pesticide application.

## 1. Introduction

The fall armyworm (FAW) or *Spodoptera frugiperda* (J.E. Smith) (Lepidoptera: Noctuidae) is a polyphagous moth pest that can feed on more than 300 host plant species [1] though has a preference for cultivated grasses, including maize, sorghum, and wheat [2]. FAW has the potential to reduce annual maize production by 21‒53% in the absence of control methods [3]. The FAW is native to the tropical and subtropical regions of the Americas [4]. Because of the globalization of trade [5] and FAW’s strong dispersal ability, the impact of this pest has extended to other continents in recent years. It was first reported in Africa in 2016 [6], then in South Asia in 2018 [7,8,9,10]. In January 2019, the FAW was first discovered in Yunnan, China, and by the end of 2019, it was recorded in 1524 counties in 26 provinces/municipalities, with a total damage area of 1125.33 thousand hectares [11]. Yunnan is currently the most severely affected area: from January to September 2019, the area affected by the FAW in Yunnan accounted for 59.31% of the total affected area in China [12]. Due to the topography of the Qinghai‒Tibet Plateau, a new generation of FAWs from Southeast Asia will fly into East Asia through Yunnan every year, and part of them will grow, develop and reproduce in Yunnan since the geographic location and environmental conditions of Yunnan are greatly beneficial for the colonization of FAW. In spring, some of these FAWs in Yunnan will disperse northward and impact other areas.

Unlike many migratory insect pests, FAW does not have the ability to diapause [13], and cannot, therefore, withstand severe cold. Northern outbreaks of FAW start with reproduction in tropical and subtropical regions and disperse northward in the spring [14]. In the United States, FAW can only overwinter in South Florida and Texas [15], i.e., south of about 28° N. It is suggested that climate, especially temperature, is critical for range expansion of FAW [16]. During the winter, the life cycle of FAW is about 80–90 days, and the pupal stage is about 20–30 days, which is longer than for other seasons [14,17]. In addition, the effective overwintering area also depends on the winter planting of the host crop [12] since the host crop is both the main food source and habitat of FAW. The soil has an impact on the growth of both FAW and host plants. The pupation of FAW usually occurs in the surface and shallow soil [18]. Experiments in Florida, USA, showed that the rate of adult emergence during the overwintering period was positively correlated with the average soil temperature [19]. The interaction between soil type and environment (such as precipitation, temperature, and moisture) also significantly affects pupation and the adult emergence rate [15,20]. The synergistic and independent response of environmental factors in terms of the suitability of conditions that favor FAW growth and propagation deserves attention. The outbreak of FAW is largely dependent on the prevailing environmental conditions in the overwinter months [14,15]. Studying the potential overwintering distribution where the environment condition is suitable for the survival of FAWs during the winter period is the key to understanding the scale of the insect source and making early management decisions.

The use of ecological niche models (ENMs) is currently an effective method for predicting the potential distribution of invasive species, mainly including the process-based (PB) and niche-based (NB) models [21]. The PB model requires accurate eco-physiological response data for a species to predict its distribution, but these data are often lacking, while the NB model needs more easily available species existence data [22], which also makes the NB model more effective and commonly used than the PB model. NB models include artificial neural networks (ANN), the maximum entropy method (MaxEnt), the genetic algorithm for rule-set production (GARP), BIOCLIM, and random forest (RF) [23]. Among these, MaxEnt is widely used because of its flexibility and performance [24,25,26]. In MaxEnt, the occurrence of the species and the environmental variables should be provided, and the target distribution is estimated by finding the probability distribution of maximum entropy [27]. MaxEnt has been used to explore the potential distribution of FAW [5,28,29]. Most of these studies focused on the influence of climate on the suitability of FAW and made long-term predictions. However, pests and diseases are highly dynamic in terms of time and space [30]. This study hopes to make short-term predictions of the potential distribution of FAW during the overwintering period, which also has higher requirements in terms of the environmental data. Compared with interpolated meteorological data, remote sensing provides a data source with higher spatial and temporal resolution [31] and can describe the real-time local environmental conditions in detail. Kumbula et al. [32] used remote sensing data provided by the Sentinel-2 MSI sensor to study the potential area of wood borer pest *Coryphodema tristis* occurrence; the results showed that high-precision prediction results could be obtained from the use of remote sensing data and vegetation indices. Malahlela et al. [33] and Truong et al. [34] used remote sensing data to predict the potential distribution of invasive species; compared with using only climate data, the results showed that the predicted area of suitable habitat is closer to the actual situation without any decrease in accuracy. In most cases, adding remote sensing data can improve the accuracy of SDM models [31].

In this study, we used the high spatial-temporal resolution data provided by the remote sensing and assimilation system and considered the three environmental factors of meteorology, vegetation, and soil, aiming to (1) select the MaxEnt model with the best combination of parameters to predict the potential distribution of FAW during the overwintering period (January to March) in Yunnan, (2) analyze the changing trends of the potential overwintering distribution of the FAW, and (3) explore the response and relative contribution of environmental factors to FAW’s suitability. The results of the study can provide a basis for the early prevention and control of the FAW by guiding management and decision-making, and may facilitate reductions in pesticide application.

## 2. Materials and Methods

### 2.1. Study Area and Species Occurrence Record

**Figure 1:**
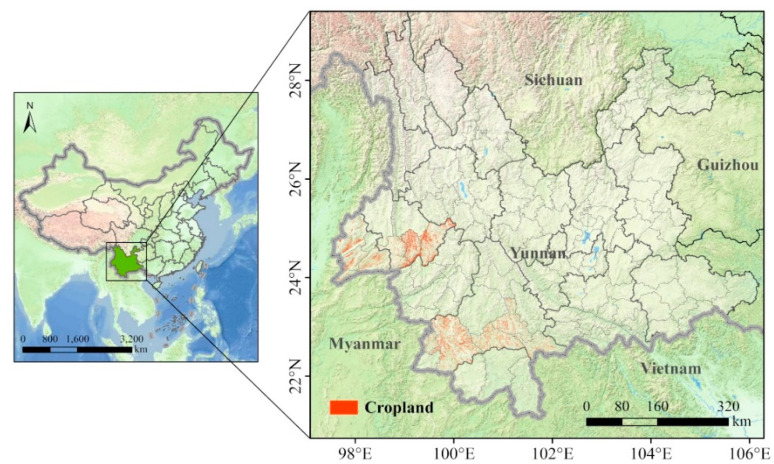
Study area and cropland in regions with recorded fall armyworm (FAW) occurrence.

The study area is located in Yunnan Province (97°31′‒106°11′ E, 21°8′‒29°15′ N), in southwestern China, with an area of approximately 3.9 × 10^5^ km^2^. The highest altitude in Yunnan is 6740 m and the lowest is 76.4 m. The average temperature of the coldest month (January) is 6–8 °C, and the daily temperature difference can reach 12–20 °C in winter and spring. The occurrence records of the FAW in January 2019 were obtained from the National Agro-Tech Extension and Service Center (https://www.natesc.org.cn/). To obtain more precise geographic coordinates for the occurrence of FAW in consideration of the huge damage it caused to cropland in these regions, we used cropland data (including Rainfed Cropland and Herbaceous Cover) of occurrence regions for further study (Figure 1). Cropland data were obtained from Fine Land-Cover Mapping in China [35], which can be downloaded from CASEarth (http://data.casearth.cn/). To reduce spatial autocorrelations, cropland data were spatially rarefied with a radius of 10 km using SDMtoolbox [36,37].

### 2.2. Environmental Factors

This research used three types of environmental factors, including meteorology, vegetation, and soil. To characterize the dynamics of environmental variables, monthly data from January to March 2019 were used in the research. To avoid strong collinearity between variables, we retained the variables with Pearson’s correlation coefficient < 0.9 (Figure S1). Meteorological factors, including the average 2 m air temperature, total precipitation, and average humidity data [38], were obtained from the National Earth System Science Data Center (http://www.geodata.cn/). MODIS MOD13A2 V6 products [39], including the normalized difference vegetation index (NDVI) and the enhanced vegetation index (EVI), were used to describe the vegetation conditions. We downloaded MOD13A2 V6 products from Google Earth Engine (https://earthengine.google.com/). Monthly soil factors including the 0‒10 cm average soil moisture and average soil temperature were derived from CLDAS V2.0 products [40]; we downloaded them from China Meteorological Data Service Center (http://data.cma.cn/). Moreover, in this research, we also considered three nonmonthly soil factors, including soil type, 0‒5 cm silt content, and 0‒5 cm clay content [41], which showed no significant differences between months but also affect the suitability of conditions for FAW; we downloaded them from Soilgrids (https://www.soilgrids.org/). All of the environmental factors used in this study (Table 1) were at a spatial resolution of 1 km × 1 km or were resampled to this resolution by using the nearest-neighbor method.

### 2.3. MaxEnt Modeling

MaxEnt [42] version 3.4.1 (biodiversityinformatics.amnh.org/open_source/maxent) was used to explore the potential overwintering distribution of the FAW. As the presence-only model, MaxEnt has good predictive performance at modeling the niche of species [43]. The general MaxEnt model formula is as follows:

(1) $$P_{w}\left(y|x\right)=\frac{1}{Z_{w}\left(x\right)}\exp\left(\sum_{i=1}^{n}w_{i}f_{i}\left(x,y\right)\right)$$

(2) $$Z_{w}\left(x\right)=\sum_{y}\exp\left(\sum_{i=1}^{n}w_{i}f_{i}\left(x,y\right)\right)$$

where *x* is the input environmental variable, *y* is the cropland location of the FAW occurrence regions, $f_{i}\left(x,y\right)$ is the characteristic function, $w_{i}$ is the weight of the characteristic function, *n* represents the number of datasets, and $P_{w}\left(y|x\right)$ is the output, which represents the suitability for FAW [44]. 

Moreover, we chose “subsample” as the replicated run type, and 70% of the sample points were randomly selected for training while the remaining 30% were set aside for testing. The convergence threshold was set to 10^−5^. When the log loss per iteration dropped below the convergence threshold, the training would stop. The output format chosen for predicted distributions was logistic, where the values are probabilities (between 0 and 1) which can be interpreted as relative suitability [45]. The prediction performance and model complexity of MaxEnt is affected by feature types and regularization parameters [46]. In order to have the best prediction performance and avoid model overfitting, we tested models for all combinations of the following feature types and regularization parameters: L (linear), LQ (linear and quadratic), H (hinge), LQH (linear, quadratic, and hinge), and LQHP (linear, quadratic, hinge, and product) and regularization multipliers from 0 to 5 in increments of 0.5 for a total of 50 combinations.

### 2.4. MaxEnt Validation

To select the optimal model, we used SDMtoolbox [37] to evaluate the model. Previous studies often used the area under the receiver operating characteristic curve (ROC) of the test data, i.e., AUC, to evaluate the quality of the models. The range of AUC values was 0.5–1. A random model has an AUC of 0.5, and a perfect model has an AUC of 1 [27]. In fact, it is not the case that the higher the AUC, the better the model. Model overfitting may also lead to higher AUC. The omission rate can quantify overfitting [46]. We hope that the model had both a low omission rate (OR) and a high AUC. Therefore, we used the prediction rate (PR, 1 − OR) + AUC as the evaluation index to select the optimal model [37]. If the models had the same PR + AUC, we chose the model with the lowest complexity of model feature classes. The order of complexity of model feature classes, from low to high, was L, LQ, H, LQH, LQHP [37,46]. Meanwhile, the “Create response curve” and “Do jackknife” options were selected to evaluate environmental variables.

## 3. Results

### 3.1. Model Optimal Parameter Evaluation

This study used PR + AUC as the evaluation index to select the optimal MaxEnt model parameters combination for predicting the overwintering potential distribution of the FAW. The model with the largest PR + AUC was considered the optimal model. The result of each parameter combination was the average of 10 replicates. Among the 50 models (Figure S2), when the feature class was LQH and the regular parameter was 1, the model worked best, with PR of 0.897 and AUC of 0.848.

### 3.2. Potential Overwintering Distribution

**Figure 2:**
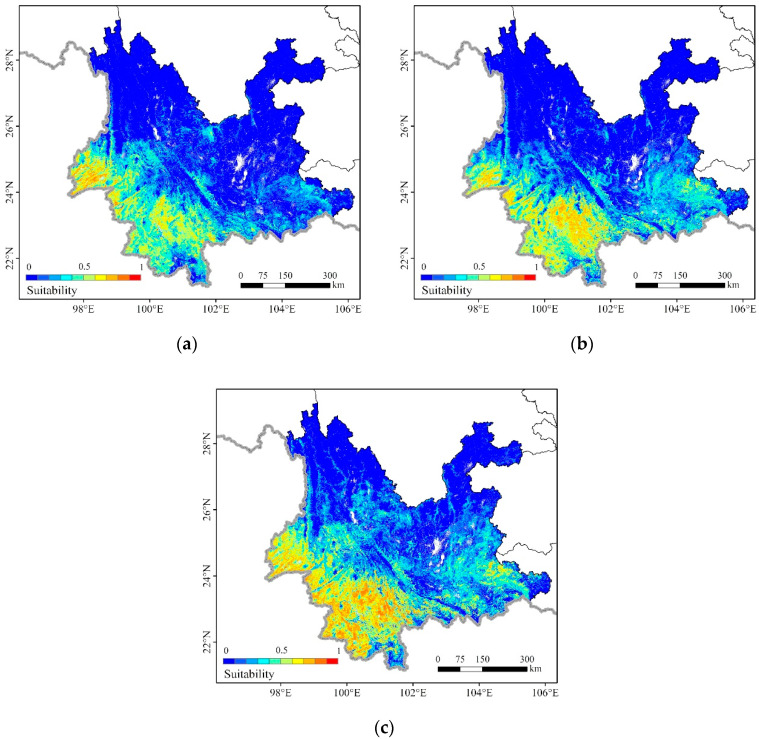
The MaxEnt model with optimal combinations of feature classes and a regularization multiplier to predict the potential overwintering distribution of the FAW from January to March 2019. (**a**) January; (**b**) February; (**c**) March.

Using the optimal model to predict the potential distribution of the FAW from January to March, the prediction results are shown in Figure 2. Suitability (the average logistic output) ranges from 0 to 1, where 0 means unsuitable and closer to 1 means more suitable for FAW survival. The results showed that the model could accurately predict the potential distribution of the FAW in accordance with the survey conducted by the National Agricultural Technology Extension Service Center. In January, FAW was found in 12 new counties and the average suitability of the occurrence areas was 0.498. In February, FAW was discovered in 8 new counties and the average suitability of the occurrence areas was 0.428. In March, FAW was observed in 18 new counties and the average suitability of the occurrence areas was 0.447. 

**Figure 3:**
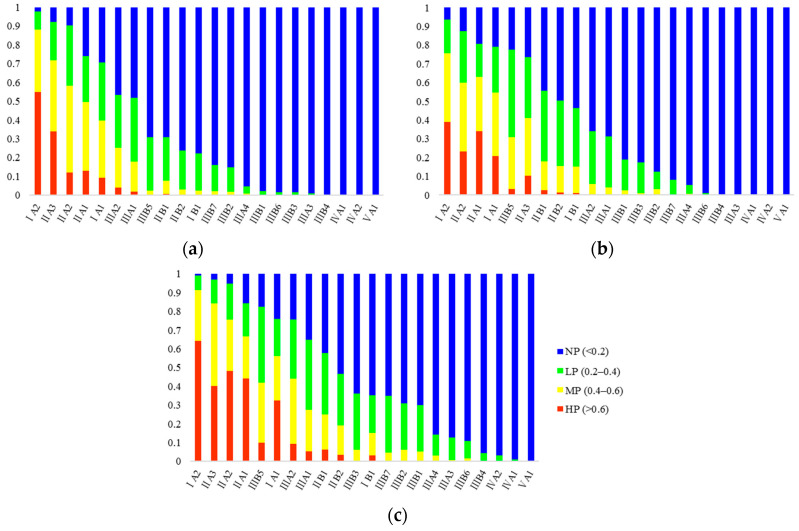
A stacked bar chart of 22 integrated natural zones in Yunnan showing the proportions of the four suitability categories. Sorted by NP from low to high: (**a**) January; (**b**) February; (**c**) March.

To quantitatively understand the potential distribution of FAW, the degree of suitability of each region was classified into four levels: high potential (HP) (>0.6), moderate potential (MP) (0.4–0.6), low potential (LP) (0.2–0.4), and no potential (NP) (<0.2) [47], and zonal statistics for the proportions of the four types of suitability were conducted for 22 integrated natural zones in Yunnan (Figure S3). The integrated natural zones fully consider the connection and specificity of the agricultural conditions, natural resources, and the natural geographical environment in various regions of Yunnan and form an important basis for guiding agricultural production. We sorted the 22 integrated natural zones according to the proportion of NP from low to high (Figure 3).

According to the ranking results, IA2 (Dehong, Mengding mid-mountains and strath district), IIA3 (Lianghe, Longling mid-mountains and plateau district), and IIA2 (Lincang mid-mountains and plateau district) were the top three in January. IA2′s HP accounted for 55.0%, 38.9%, and 64.3%, respectively, from January to March. IIA3′s HP dropped by 23.4% in February, while it rose in March with a rise of 29.7%. From January to March, IIA2′s HP accounted for 12.0%, 23.3%, and 48.2%, respectively, showing a continuous upward trend. IIA1 (Simao mid-mountain, plateau and basin district), IA1 (Xishuangbanna middle-low mountains, basin and strath district), located in southern and central areas of Yunnan, have similar trends to IIA2. The NP of IB1 (Hekou mid-mountains and low-valley district), IIB1 (Mengzi, Yuanjiang plateau, basin, and canyon district), IIB2 (Wenshan karst mountains and plateau district), and IIIB5 (Qiubei, Guangnan karst mountains and plateau district) in western Yunnan dropped by 24.1%, 24.9%, 26.6%, and 46.7%, respectively, in February. In March, the NP of IIIA2 (Tengchong middle-low mountains, basin and strath district), IIIA1 (Baoshan, Fengqing middle-low mountains, basin and strath district), IIIB7 (Jinsha river valley district), IIIB2 (Chuxiong, Hongyan plateau district), IIIB1 (Kunming, Yuxi lake, basin and plateau district), and IIIB3 (Qujing Karst and Plateau District) in central and northern Yunnan all decreased. From January to March, the HP of IVA1 (along the Northeast of Yunnan mid-mountains and river valley district), IVA2 (Zhengxiong mid-mountains and plateau district), and VA1 (The Northwest Yunnan high-mountain and plateau district) were close to 0%, and the NP was higher than 95%.

**Figure 4:**
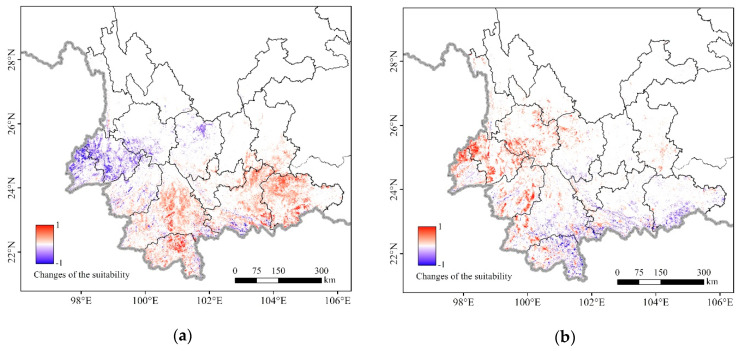
Changes in the suitability for FAW between months: (**a**) January to February; (**b**) February to March. Indicates the suitability has increased (red) or decreased (blue).

According to the independent sample *t*-test results, there was a significant difference in suitability from January to March (*p* < 0.05). The mean of suitability was 0.179 in January, 0.205 in February, and 0.265 in March. The suitability gradually rose from January to March, but in some areas, the suitability declined in February, especially in western Yunnan. This may be due to the local temperature drop in western Yunnan, indicating that local suitability is sensitive to the fluctuation of local environmental factors over a short period of time. However, with the warming of the weather, the beginning of spring planting, and the emergence of seedlings, the level of suitability in March rose above that of January. The change in the suitability of the FAW (Figure 4) also confirmed this point. In general, during the overwintering period, the area most likely to be affected were concentrated in the southwest of Yunnan; their suitability continued to increase in addition to gradually extending to eastern Yunnan and a few northern areas. Yunnan has a complex topography and climatic differences. Although Yunnan is widely recognized as the annual breeding area of FAW, not all of its regions are suitable for FAW to overwinter due to the temporal and spatial differences in environmental factors.

### 3.3. Factors Shaping the Potential Overwintering Distribution of FAW

#### 3.3.1. Environmental Variable Importance Analysis

We used two types of data, i.e., monthly and nonmonthly, to accurately reflect the areas suitable for FAW in monthly intervals. The results (Table 1) showed that the percent contribution of monthly data reached 50.5%, which also indicated that these monthly data are important for short-term prediction. Among the monthly data, meteorological factors accounted for 30.6%, followed by vegetation factors (16.5%) and soil factors (3.4%). Although the importance of monthly soil factors was relatively low, this does not mean that soil factors have little effect on the prediction. In fact, nonmonthly soil factors such as soil type, silt content, and clay content do not show a significant change from month to month but are crucial for FAW, which has many physiological activities in the surface and shallow soil. The percent contributions of soil classification, silt content, and clay content were 21.4%, 25.7%, and 2.4%, respectively. Moreover, judging from the results of the jackknife test of each variable importance, the environmental variable with the highest training gain when used in isolation was an average 2 m air temperature (0.50), followed by silt content (0.46), EVI (enhanced vegetation index, 0.43), and average humidity (0.41). The lowest independent training gain was observed for average 0‒10 cm soil moisture (0.04).

#### 3.3.2. Response Curves of the Top Four Environmental Variables

**Figure 5:**
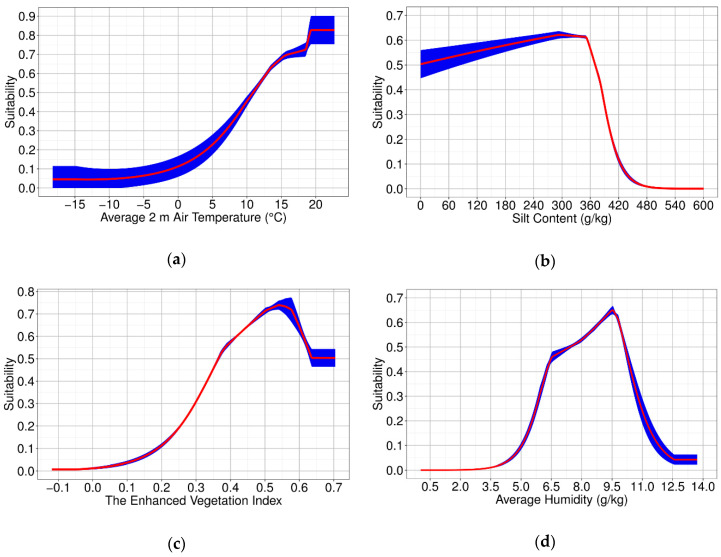
Response curves of the top four environmental variables. The curves show the average response of the 10 replicate Maxent runs and the mean (red) ± one standard deviation (blue) for (**a**) average temperature; (**b**) silt content; (**c**) EVI; (**d**) average humidity.

The four environmental factors with the highest independent training gain were average temperature, silt content, EVI, and average humidity. The training gains were all greater than 0.4. The response curves of the top four environmental factors are shown in Figure 5. With the increase in the average 2 m air temperature, the suitability continued to rise. At 18.45 °C, there was a slight fluctuation whereby the growth rate of suitability increased. At 19.35 °C, the average adaptability reached a peak of 0.83. When the silt content was between 0 and 295.28 g/kg, the suitability continued to increase, and the highest value of average suitability was 0.62, after which the suitability decreased slightly. In the range of 347.69 to 544.25 g/kg, the suitability dropped rapidly and eventually tended to 0. As for EVI, when EVI was less than 0.39, the suitability rose rapidly. Subsequently, the suitability growth rate slowed down, and when EVI reached 0.54, the average suitability peaked at 0.740. After 0.54, the suitability began to decline, and when EVI was 0.64, the suitability was stable with an average value of 0.50. The suitability started to increase when the average humidity was 1.18 g/kg and reached the response peak at 9.53 g/kg, with maximum average suitability of 0.65; then, the suitability declined with an increase in average humidity, and at 12.59 g/kg, the suitability was less than 0.05. Comprehensive consideration of environmental factors is an effective way to improve the model’s predictive ability. Through the response curves, we can study the changes in overwintering suitability of the FAW in Yunnan with each variable. 

## 4. Discussion

FAW is a major transboundary migratory insect pest about which the United Nations Food and Agriculture Organization has issued alerts. FAW, which has invaded Asia, including China, is a maize strain with some heterozygotes, and mainly harms crops such as corn, sugar beet, and wheat [2], threatening more than 50% of China’s main corn grain-producing areas. The biological invasion is then succeeded by large-scale outbreaks of pests in the next few years. FAW invasions pose hidden dangers through future reproduction and damage in China and even the rest of East Asia. At present, the Chinese government has launched the “three areas, three belts, and three lines of defense” measures to deal with FAW. According to the dispersal path of FAW, the cultivation situation of host crops, and meteorological conditions, the task of preventing the FAW invasion has been arranged in 205 key counties in 17 provinces. As the primary prevention and control site in the first line of defense, Yunnan is affected by the superimposed influence of foreign and local sources of FAW. The presence of FAW in Yunnan has the characteristics of earlier occurrence, wider range, and severe damage.

This study predicted the potential distribution of FAW from January to March 2019 in Yunnan. Although most areas of Yunnan are in the tropics and subtropics, the regional differences and vertical changes in Yunnan’s climate are very stark. Not all areas are suitable for the growth of the host plant and the overwintering of FAW. The results show that the changes in FAW’s potential overwintering distribution are not limited to the expansion of the area, but also improvement of the degree. During the overwintering period, the area most likely to be affected are concentrated in the southwest of Yunnan, then gradually extend to eastern Yunnan and a few northern areas, and the suitability level gradually increases from January to March. However, in some areas, especially western Yunnan, the suitability of the FAW declines from January to February, but rebounds from February to March, which shows that local suitability is sensitive to fluctuations in local environmental factors over a short period of time.

In order to comprehensively consider the impact of environmental factors, this study used a total of 10 environmental factors corresponding to three categories: meteorological, vegetation, and soil factors. Most previous studies have mainly considered the impact of meteorological factors on the suitability for FAW. In fact, this study believes that meteorology is an important factor affecting the suitability for FAW, but not the only factor. The influence of vegetation and soil cannot be ignored. In terms of the monthly percent contribution, meteorological factors accounted for 30.6%, followed by vegetation factors at 16.5%, and soil factors accounted for 3.4%. In addition, the percent contributions of nonmonthly soil data from soil classification, silt content, and clay content were 21.4%, 25.7%, and 2.4%, respectively. The dry season in Yunnan is from November to April of the following year, during which the precipitation only accounts for 15% of the whole year, and our research time range is included in the dry season. Nboyine et al. [48] believe that rainfall will first lead to the prosperous growth of host plants, thereby creating conditions for the growth of the FAW population; at the same time, rainfall and irrigation will wash away or drown FAW. From the response curve of total precipitation (Figure S4), the precipitation during the overwintering period did not reach the turning point at which the suitability for FAW began to decline. In more fragile soils, rainfall will cause the soil to collapse [20]. This is consistent with the response curve of silt content. When the silt content is greater than 347.69 g/kg, the suitability shows a rapid downward trend. Plessis’s [13] research suggests that the optimum temperature for FAW differs in each growth period, ranging from 26 to 30 °C. From the response curve of average 2 m air temperature, the suitability increases with the increase in temperature, and during the overwintering period, the average 2 m air temperature did not reach the temperature optimum for FAW. Host plants are an important habitat and food source of FAW. Westbrook et al. [49] used the time and place of corn planting, combined with meteorological conditions, to simulate the multigenerational migration of FAW in the USA. The type and spatial distribution of host plants are closely related to the occurrence and distribution of FAW [50,51,52,53]. Corn is widely planted during the wintering period in Yunnan, which is the main growing season, providing sufficient food resources for FAW. This study uses NDVI and EVI to reflect current crop growth. From the results of independent gain training, EVI has a higher training gain than NDVI, which may be because EVI can maintain sensitivity over dense vegetation conditions. Judging from the response curves of the two vegetation indices, the suitability is reduced beyond a certain threshold. As FAW mainly harms crops, areas with a higher vegetation index are more likely to be forests, resulting in decreased suitability [4]. 

Remote sensing data provide the possibility of real-time monitoring and short-term prediction of FAW distribution. This study used MODIS remote sensing products and datasets from assimilation systems such as CLDAS V2.0 and Soilgrids as the main data sources to obtain the monthly environmental conditions from January to March 2019. The addition of remote sensing data and products has improved the spatial and temporal resolution of the potential distribution and made it more accurate. At present, remote sensing has been widely used in the monitoring and prediction of plant diseases and pests [54]. We also believe that remote sensing will be more effective in the future prevention and control of FAW, for example, by monitoring the severity of damage caused by pests based on changes in the biomass of damaged crops [55] or narrowing the scope of control through remote sensing inversion of host plants. However, the fragmented land in Yunnan and scattered planting by farmers have resulted in large variations in the growth period of crops. Meanwhile, cloud and foggy weather in Yunnan have also increased the difficulty of crop remote sensing inversion. Under the current circumstances, the use of the vegetation index is a relatively convenient and efficient way to reflect vegetation conditions.

## 5. Conclusions

FAW is a long-distance migratory insect pest that is currently affecting more than 100 countries and regions. Yunnan is one of the sites where insect sources from Burma, Vietnam, and Laos invade China and East Asia, and its geographic location and climatic conditions are favorable for FAW overwintering. In this research, we studied the potential distribution of FAW in Yunnan through cropland in areas where the FAW has occurred during the overwintering period, aiming to learn and train the known environmental background and to summarize the environmental laws governing occurrence. In order to obtain more reliable potential distribution results, we used the MaxEnt model with good flexibility and performance in species niche modeling and screened the optimal parameter combinations. The results show that, of the 50 MaxEnt parameter combinations, when the feature class is LQH and the regular parameter is 1, the model has the highest PR + AUR with a PR of 0.897 and AUC of 0.848. The potential overwintering distribution of FAW has high temporal and spatial dynamics, and the influence of monthly data reaches 50.5%. The areas with the highest suitability of the FAW in Yunnan are concentrated in southwestern Yunnan, and its suitability continues to increase from January to March, gradually extending to eastern Yunnan and some northern areas. In view of the changes in the overwintering potential distribution, we should not only pay attention to the expansion of its geographic scope, but also to the increase in severity in the same area.

In previous research, the response of the FAW has mainly been studied with respect to climate, but variables describing the surface conditions, such as vegetation and soil, have rarely been considered. In this study, the introduction of remote sensing and assimilation systems provided a data source with a higher spatial and temporal resolution which can represent real-time surface vegetation and soil conditions and can obtain more precise prediction results. The monthly independent contributions of meteorological, vegetation, and soil factors were 30.6%, 16.5%, and 3.4%, respectively, and the contribution of nonmonthly soil data was 49.5%, indicating that the suitability of FAW was not solely dominated by meteorological factors; ground surface factors also play a decisive role in determining suitability. This research could provide methodological support for early warning and efficient prevention and control of FAW, as well as achieving meaningful reductions in pesticide application and ensuring national food security.

## Figures and Tables

**Table 1:** List of the importance of the environmental variables.

Class	Variables	Percent Contribution	Training Gain with Only Variable
**Monthly Data**			
Meteorology	Average 2 m Air Temperature (°C)	1.6	0.50
	Total Precipitation (mm)	5.5	0.15
	Average Humidity (g/kg)	23.5	0.41
Vegetation	Normalized Difference Vegetation Index	10.1	0.33
	Enhanced Vegetation Index	6.4	0.43
Soil	Average 0‒10 cm Soil Moisture (m^3^/m^3^)	2.8	0.04
	Average 0‒10 cm Soil Temperature (°C)	0.6	0.30
**Nonmonthly Data**			
Soil	Soil Classification	21.4	0.29
	0‒5 cm Silt Content (g/kg)	25.7	0.46
	0‒5 cm Clay Content (g/kg)	2.4	0.17

## Supplementary Materials

The following are available online at https://www.mdpi.com/2075-4450/11/11/805/s1: Figure S1: Pearson’s correlation matrix of environmental variables, Figure S2: AUC + PR of 50 model parameter combinations, Figure S3: Map of Yunnan integrated natural zones, Figure S4: Response curves of the environmental variables.
