# Supplementary material for: Overwintering Distribution of Fall Armyworm (Spodoptera frugiperda) in Yunnan, China, and Influencing Environmental Factors
Source: Insects. 2020 Nov 15;11(11):805. doi: 10.3390/insects11110805 (PMC7696661; doi:10.3390/insects11110805)
Supplement: Supplementary file 1 [file insects-11-00805-s001.pdf]

Supplementary Materials

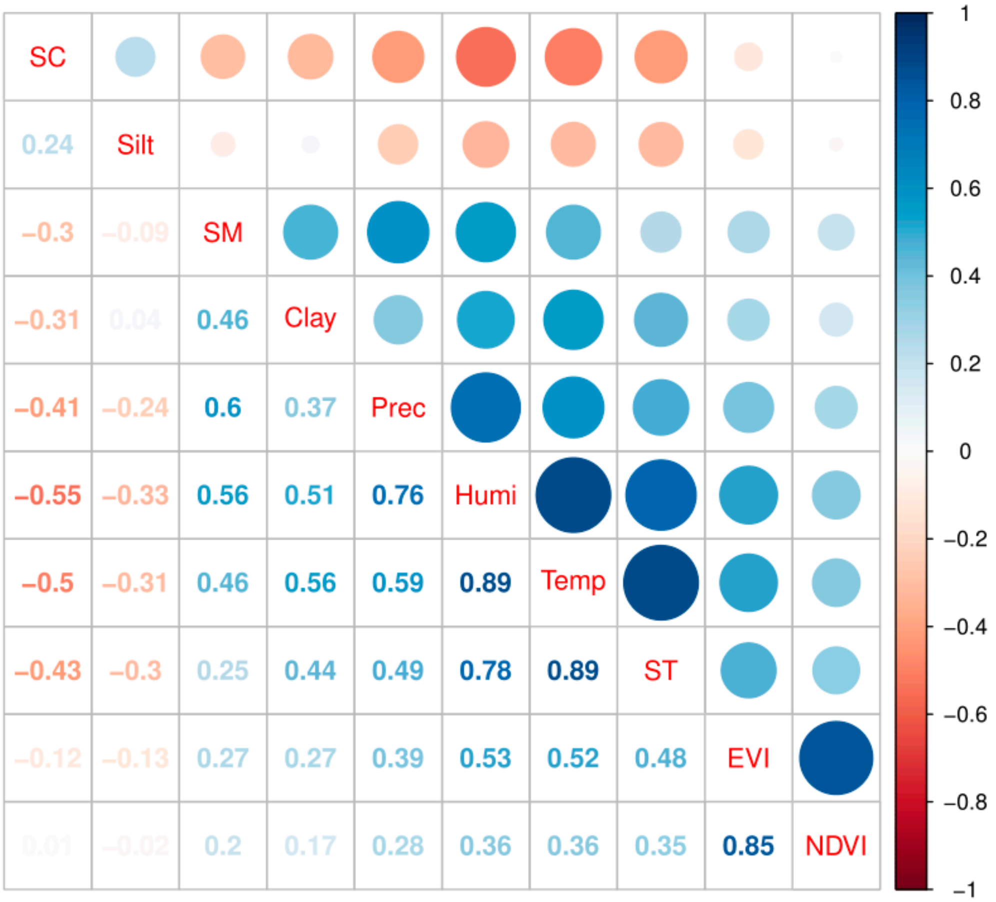

Figure S1. Pearson's correlation matrix of environmental variables.

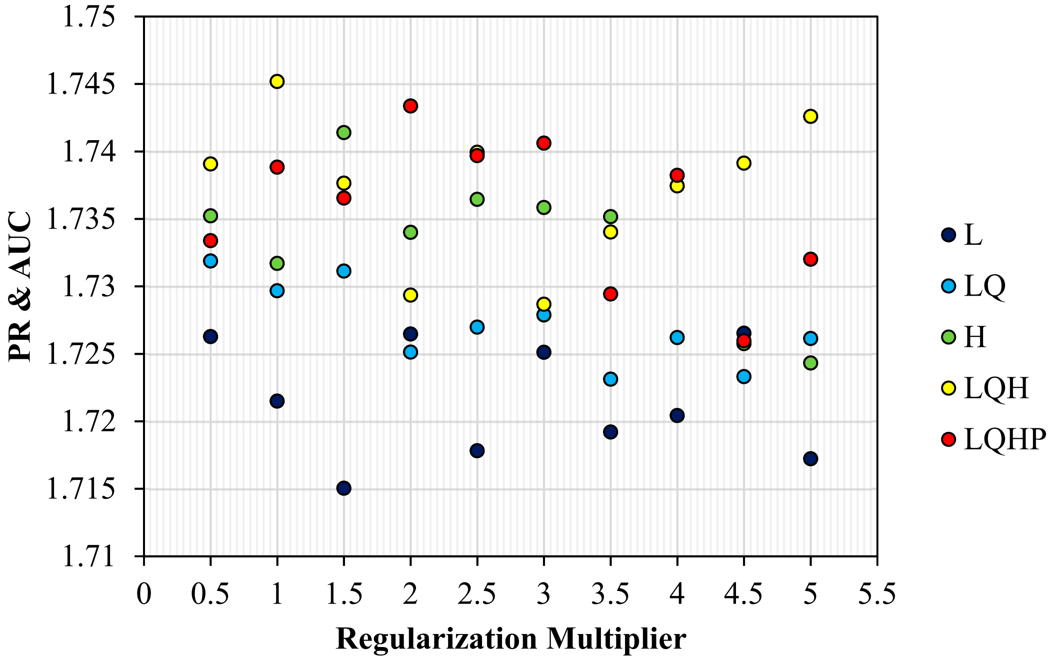

Figure S2. AUC + PR of 50 model parameter combinations.

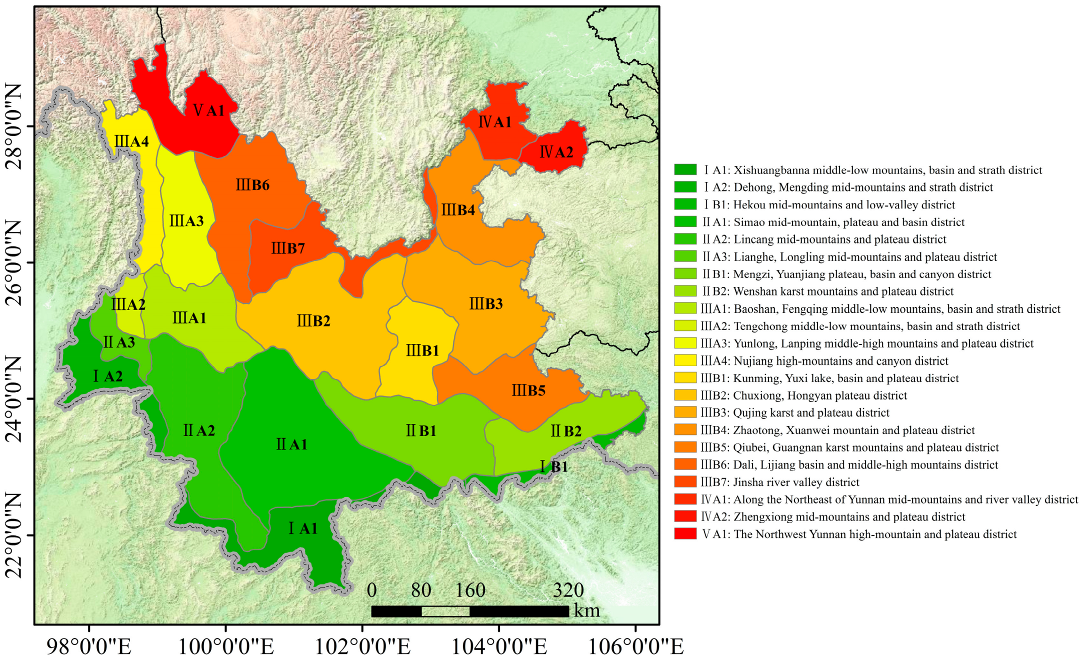

Figure S3. Map of Yunnan integrated natural zones.

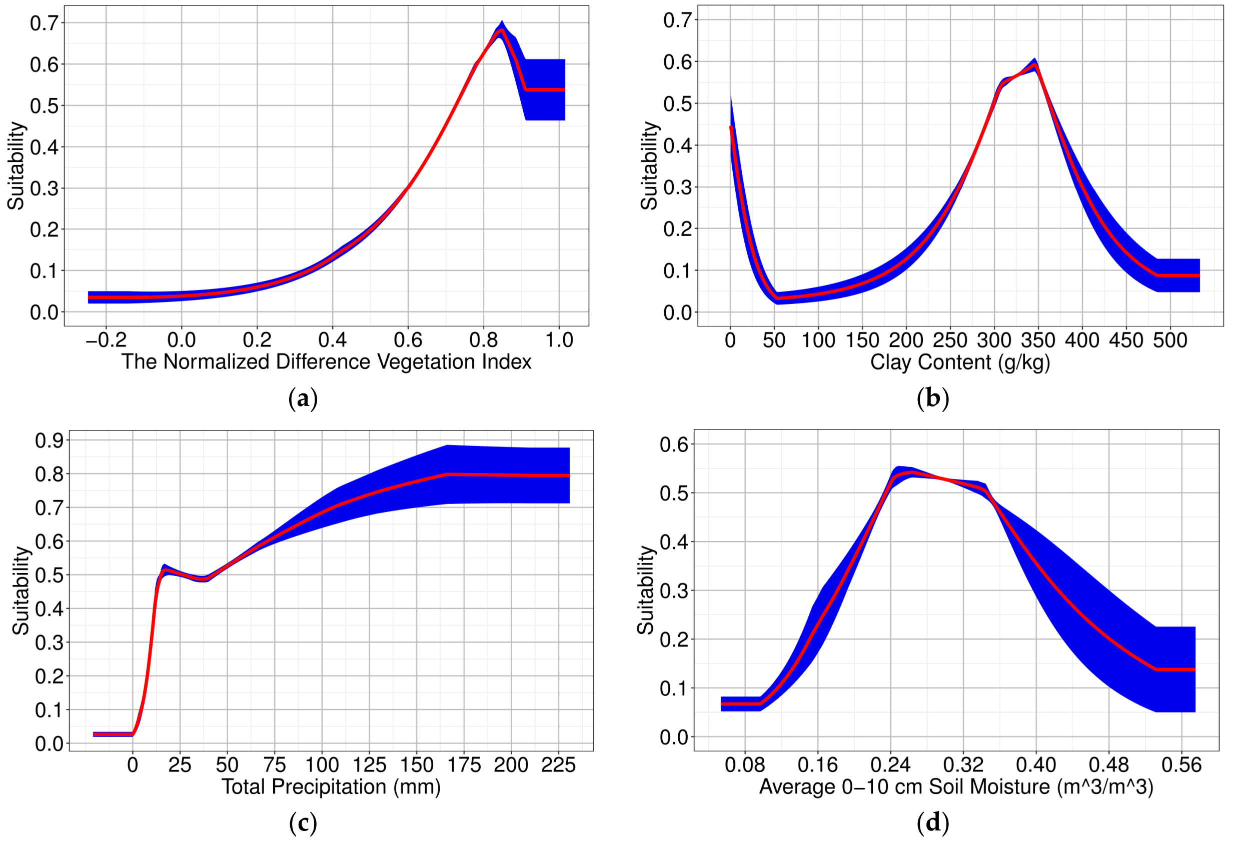

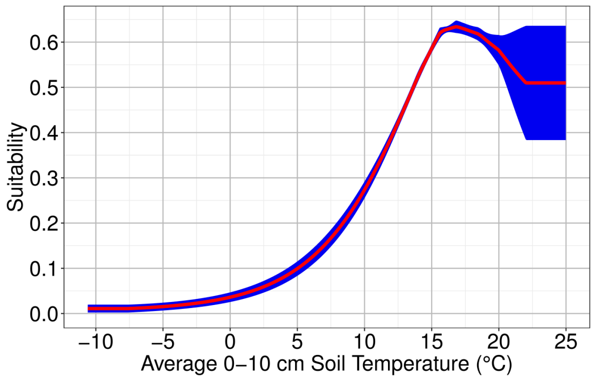

(e)

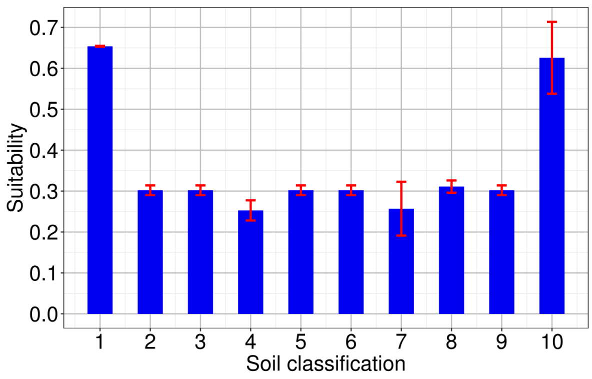

(f)

Figure S4. Response curves of the environmental variables. The curves show the average response of the 10 replicate Maxent runs and the mean ± one standard deviation. (**a**) NDVI; (**b**) 0–10 cm soil moisture; (**c**) 0–10 cm soil temperature; (**d**) Soil classification (1: Acrisols; 2: Alisols; 3: Andosols; 4: Cambisols; 5: Ferralsols; 6: Fluvisols; 7: Leptosols; 8: Luvisols; 9: Regosols; 10: Vertisols).
